# Exploring the Pharmacological Potential of *Onosma riedliana*: Phenolic Compounds and Their Biological Activities

**DOI:** 10.1007/s11130-023-01131-0

**Published:** 2023-12-16

**Authors:** Sanja Ćavar Zeljkovıć, Saliha Seyma Sahinler, Cengiz Sarikurkcu, Bulent Kirkan, Riza Binzet, Petr Tarkowski

**Affiliations:** 1 Czech Advanced Technology and Research Institute, Palacky University, Šlechtitelů 27, Olomouc, 78371 Czech Republic; 2 Centre of the Region Haná for Biotechnological and Agricultural Research, Department of Genetic Re-sources for Vegetables, Medicinal and Special Plants, Crop Research Institute, Šlechtitelů 29, Olomouc, 78371 Czech Republic; 3 Faculty of Pharmacy, Afyonkarahisar Health Sciences University, Afyonkarahisar, TR-03100 Turkey; 4 Faculty of Arts and Science, Department of Biology, Mersin University, Mersin, TR-33343 Turkey

**Keywords:** *Onosma Riedliana*, Phenolic Compounds, Antioxidant Activity, Enzyme Inhibitory Activity

## Abstract

*Onosma riedliana* Binzet & Orcan, a traditionally used plant species, has been explored for its therapeutic potential in this study. The work presented here is the first report on the phenolic profile and biological activity of this species. Three extracts of varying polarity were prepared, with the methanolic extract containing the highest phenolic content (97.62 ± 0.20 mgGAE/g). Key phenolic compounds identified included pinoresinol, hesperidin, 4-hydroxybenzoic acid, and *p*-coumaric acid. The methanolic extract exhibited exceptional antioxidant properties, rivaling Trolox as a positive control, primarily attributed to hesperidin and luteolin. Moreover, the ethyl acetate extract demonstrated remarkable inhibition of cholinesterase and tyrosinase enzymes, while the methanolic extract displayed potent activity against carbohydrate hydrolytic enzymes, α-amylase and α-glucosidase. Again, phenolic compounds were shown to be responsible for the inhibition of cholinesterases and tyrosinase, but not for α-amylase and α-glucosidase. These findings underscore *Onosma riedliana*’s potential for incorporation into diverse pharmaceutical formulations, given its multifaceted bioactivity.

**Supplementary Information:**

The online version contains supplementary material available at 10.1007/s11130-023-01131-0.

## Introduction

Throughout history, medicinal and aromatic plants have played a crucial role in treating a wide range of disorders. Phenolic compounds are found to be the major carriers of plants therapeutic properties. Numerous studies have highlighted the protective role of plant phenolics in degenerative diseases, including cardiovascular issues, cancer, diabetes, inflammation, and more [1]. The *Onosma* genus comprises approximately 150 species distributed worldwide [2]. They predominantly thrive in arid, sun-drenched environments with rocky, sandy substrates, showcasing their resilience to heat and drought. Consequently, these species tend to accumulate higher levels of phenolic compounds, aiding their survival in challenging conditions [3]. Traditionally, *Onosma* species have been used to treat various disorders, including bronchitis, abdominal pain, fever, and skin burns. Additionally, they serve as a source of red dye from the roots, commonly employed to color food items, oils, and medicinal preparations [4]. Several *Onosma* species have undergone phytochemical analysis, revealing their richness in various phenolic compounds, particularly flavonoids like luteolin and hesperidin [5–16]. Continuing the phytochemical investigation of *Onosma* species, this study represents the first report on the phenolic profiles, antioxidant properties, and enzyme inhibitory activities of three extracts derived from *Onosma riedliana*. Although *O. riedliana* was initially described by Binzet and Orcan [2], it remains relatively unexplored in the literature. Given its membership in a genus with a rich history of therapeutic use, further phytochemical exploration is warranted to unlock its pharmaceutical potential.

## Materials and Methods

The materials and methods section is presented as supplementary material.

## Results and Discussion

### Chemical Composition

**Fig. 1:**
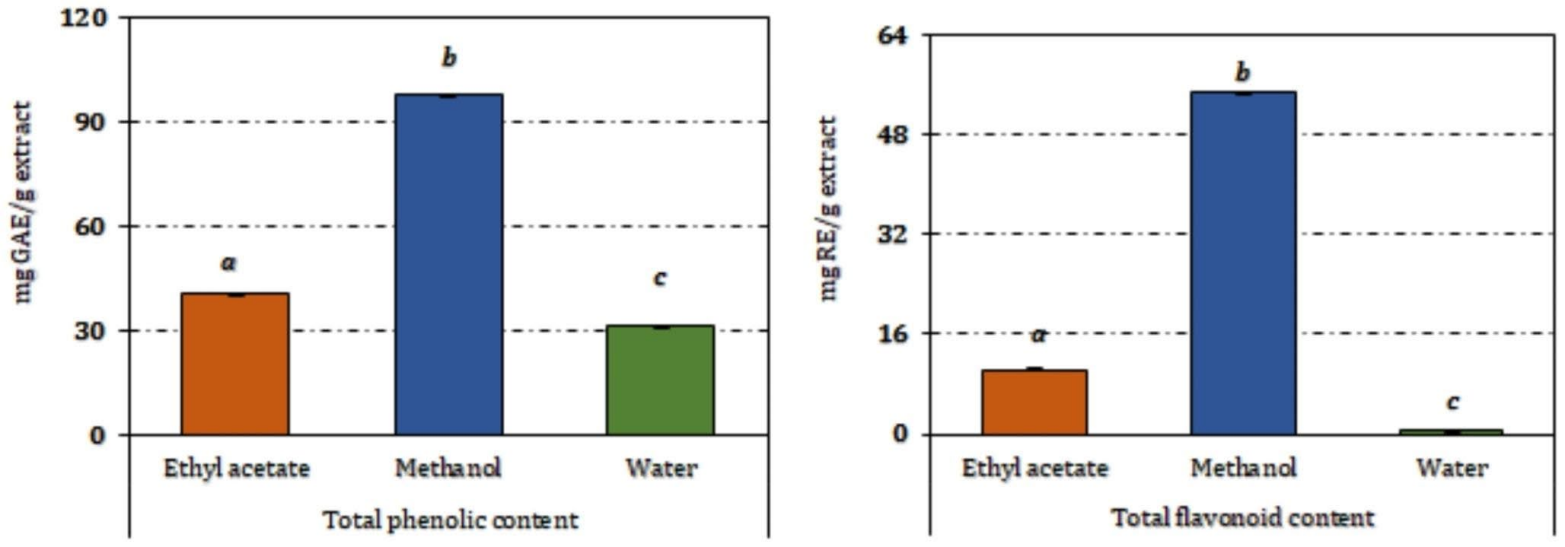
Total phenolic and flavonoid contents of *Onosma riedliana* extracts. Values indicated by the same superscripts do not significantly differ after Tukey’s *hoc* test at a 5% significance level

The yields of extracts obtained from *O. riedliana* varied among the three solvents used, with the ethyl acetate extract yielding 2.03%, the methanolic extract 0.41%, and the aqueous extract 9.28%. The selection of solvents for extracting active compounds from medicinal and aromatic plants depends on several factors, including the plant type, the specific plant part to be extracted, and the nature of the bioactive compounds. To capture a broader spectrum of phenolic compounds, this study employed three solvents with varying polarities. As per the spectrophotometric assessments of total phenolic and flavonoid content in *O. riedliana* extracts (Fig. 1), the methanolic extract exhibited significantly higher levels of these compounds compared to the other two extracts. Specifically, the methanolic extract contained 97.62 ± 0.20 mg GAE/g of phenolic compounds and 54.98 ± 0.05 mg RE/g of flavonoids (Fig. 1). In comparison to other *Onosma* species, *O. riedliana* showcased similar levels of both phenolic and flavonoid compounds as *O. aucheriana* [18], yet significantly higher levels than in other species within the genus [5–16], where the total phenolic content did not exceed 70 mg GAE/g of extract.

The detailed phenolic composition of the plant extracts was analyzed using LC-MS/MS, and the results are summarized in Table 1. The predominant compound in both the ethyl acetate and methanolic extracts was the tetrahydrofuran lignan pinoresinol, with levels of 2992.0 ± 38.9 and 73628.2 ± 1256.9 μg/g of extract, respectively. This phenol, also found in some Brassicae vegetables, sesame seeds, and olives, possesses strong antioxidant [17] and hypoglycemic [18], and antibacterial properties [19]. It’s worth noting that while pinoresinol is present in other *Onosma* species, it is typically found at significantly lower levels. The aqueous extract contained the lowest amounts of flavonoids, whereas the methanolic extract was particularly rich in hesperidin, with a concentration of 32455.3 ± 27.7 μg/g. Hesperidin, primarily found in citrus species, also stood out as the main flavonoid in the ethyl acetate extract of *O. riedliana* (Table 1). Furthermore, other *Onosma* species, such as *O. trapezuntea* and *O. rigidum* [6], *O. nana* [7], *O. papillosa* [10], *O. stenoloba* [14], and *O. mollis* [16], have been found to contain significant levels of this flavonoid.

**Table 1:** Levels (μg/g extract) of selected phenolic compounds in *Onosma riedliana* extracts

Compound	Extract		
	Ethyl acetate	Methanol	Water
Pinoresinol	2992.0 ± 38.9^b^	73628.2 ± 1256.9^a^	144.3 ± 2.0^b^
Pyrocatechol	15.0 ± 0.1^b^	29.1 ± 0.2^a^	14.5 ± 0.4^b^
Vanillin	34.6 ± 1.3^b^	784.1 ± 14.9^a^	7.7 ± 0.9^b^
2,5-Dihydroxybenzoic acid	11.3 ± 0.1^c^	98.8 ± 0.2^b^	281.1 ± 11.6^a^
3,4-Dihydroxyphenylacetic acid	143.0 ± 3.9^a^	41.7 ± 2.3^b^	27.6 ± 0.5^c^
3-Hydroxybenzoic acid	17.5 ± 0.5^b^	68.4 ± 0.6^a^	14.7 ± 0.4^c^
4-Hydroxybenzoic acid	703.5 ± 15.8^b^	9246.7 ± 7.2^a^	278.7 ± 1.1^c^
Gallic acid	4.2 ± 0.1^b^	33.5 ± 0.8^a^	4.5 ± 0.2^b^
Protocatechuic acid	69.2 ± 0.1^c^	1910.2 ± 31.9^a^	296.6 ± 4.2^b^
Syringic acid	36.6 ± 1.1^b^	1000.2 ± 41.8^a^	56.2 ± 4.8^b^
Vanillic acid	142.1 ± 5.7^b^	1829.9 ± 65.6^a^	127.4 ± 7.8^b^
2-Hydroxycinnamic acid	2.5 ± 0.0^b^	4.4 ± 0.2^a^	2.2 ± 0.2^b^
Caffeic acid	46.4 ± 0.7^b^	848.8 ± 4.3^a^	20.1 ± 0.6^c^
Ferulic acid	26.2 ± 0.4^b^	657.5 ± 8.9^a^	12.8 ± 1.7^b^
Chlorogenic acid	44.0 ± 0.7^b^	7581.0 ± 77.3^a^	3.4 ± 0.1^b^
*p*-Coumaric acid	963.3 ± 5.7^b^	13001.2 ± 52.1^a^	343.3 ± 10.1^c^
Rosmarinic acid	11.0 ± 1.1^b^	815.1 ± 2.9^a^	7.7 ± 0.1^b^
Sinapic acid	7.0 ± 0.4^b^	274.8 ± 0.8^a^	5.2 ± 0.6^b^
Verbascoside	5.7 ± 0.01^b^	8.2 ± 0.0^a^	5.8 ± 0.1^b^
(-)-Epicatechin	2.2 ± 0.0^b^	6.9 ± 0.4^a^	2.5 ± 0.0^b^
(+)-Catechin	nd	11.0 ± 0.5	nd
Apigenin	42.3 ± 0.4^b^	60.4 ± 0.4^a^	nd
Apigenin 7-glucoside	nd	19.0 ± 1.0	nd
Eriodictyol	12.1 ± 0.5^b^	25.1 ± 1.3^a^	9.44 ± 0.04^b^
Hesperidin	221.6 ± 12.2^b^	32455.3 ± 27.7^a^	1.1 ± 0.2^c^
Hyperoside	20.0 ± 1.1^b^	778.6 ± 2.6^a^	1.6 ± 0.1^c^
Kaempferol	19.2 ± 0.3^b^	39.9 ± 1.0^a^	nd
Luteolin	41.3 ± 0.4^b^	257.7 ± 1.3^a^	nd
Luteolin 7-glucoside	6.4 ± 0.7^b^	354 ± 2^a^	nd
Quercetin	11.7 ± 0.1^b^	13.6 ± 0.5^a^	1.5 ± 0.0^c^
Taxifolin	7.4 ± 0.4^c^	14.9 ± 0.5^a^	9.4 ± 0.4^b^

The values indicated by the same superscripts within the same row do not differ significantly after Tukey’s *post hoc* test at a 5% significance level. nd: Not detected

### Antioxidant Activity

To assess the antioxidant potential of *O. riedliana* more comprehensively, various assays were employed, each based on distinct mechanisms of action. These included reducing stable molybdenum (phosphomolybdenum assay), ferric (FRAP assay), and cupric (CUPRAC assay) cations, as well as reducing stable radicals (DPPH and ABTS assays) and the ability to chelate ferrous ions. The results are summarized in Table 2 and expressed as IC_50_ values (mg/mL), indicating the concentration needed to scavenge, reduce, or chelate 50% of the potential oxidant. Among the *O. riedliana* extracts investigated, the methanolic extract exhibited the most potent antioxidant activity across all six assays. In many studies, different antioxidant assays relying on various reaction mechanisms can lead to challenging and inconsistent result interpretation and comparison. To address this issue, relative antioxidant capacity indices (RACI) have been introduced. These indices offer a numerical scale that integrates multiple chemical methods, enabling the comparison of antioxidant capacity without being limited to a specific mechanism. RACI values for the methanolic extract of *O. riedliana* are notably high (Table 2), falling within the range of the most potent antioxidant plant-based foods [20]. However, there are limited reports on RACI values for other *Onosma* species, which generally exhibited lower RACI values than *O. riedliana*. For instance, methanolic extracts of *O. aucheriana*, *O. frutescens*, and *O. sericea* had RACI values of -0.49, -0.34, and 0.89, respectively [13], while *O. ambigens* exhibited a value of 0.90 [14].

**Table 2:** Antioxidant activity of *Onosma riedliana* extracts

Assay	Unit		Extract		Trolox	EDTA
		Ethyl acetate	Methanol	Water		
Phosphomolybdenum	IC_50_ (mg/mL)	1.20 ± 0.02^b^	0.55 ± 0.01^a^	2.82 ± 0.01^c^	0.60 ± 0.02^a^	
	mg TE/g	498.00 ± 8.49^b^	1087.80 ± 22.91^a^	212.40 ± 0.85^c^		
CUPRAC	IC_50_ (mg/mL)	0.95 ± 0.02^c^	0.27 ± 0.01^b^	1.79 ± 0.01^d^	0.10 ± 0.00^a^	
	mg TE/g	110.05 ± 2.43^b^	380.28 ± 2.43^a^	58.17 ± 0.26^c^		
FRAP	IC_50_ (mg/mL)	1.14 ± 0.01^c^	0.20 ± 0.01^b^	1.26 ± 0.01^d^	0.05 ± 0.00^a^	
	mg TE/g	40.73 ± 0.39^b^	230.27 ± 6.71^a^	36.87 ± 0.04^b^		
DPPH	IC_50_ (mg/mL)	4.11 ± 0.03^d^	0.30 ± 0.01^b^	3.34 ± 0.05^c^	0.05 ± 0.00^a^	
	mg TE/g	13.15 ± 0.10^b^	180.11 ± 4.01^a^	16.19 ± 0.23^b^		
ABTS	IC_50_ (mg/mL)	2.66 ± 0.02^d^	0.34 ± 0.01^b^	1.09 ± 0.01^c^	0.10 ± 0.01^a^	
	mg TE/g	37.64 ± 0.30^c^	296.65 ± 1.93^a^	92.07 ± 0.53^b^		
Ferrous ion chelating	IC_50_ (mg/mL)	1.29 ± 0.01^d^	0.95 ± 0.01^b^	1.04 ± 0.01^c^		0.03 ± 0.00^a^
	mg EDTA/g	20.51 ± 0.17^a^	27.99 ± 0.35^b^	25.43 ± 0.20^b^		
RACI	-	− 0.63 ± 0.00^b^	1.23 ± 0.03^a^	− 0.61 ± 0.01^b^		

The values indicated by the same superscripts within the same row do not differ significantly after Tukey’s *post hoc* test at a 5% significance level. IC_50_, the concentration of the extract required to reduce or scavenge or chelate 50% of potential oxidant

**Fig. 2:**
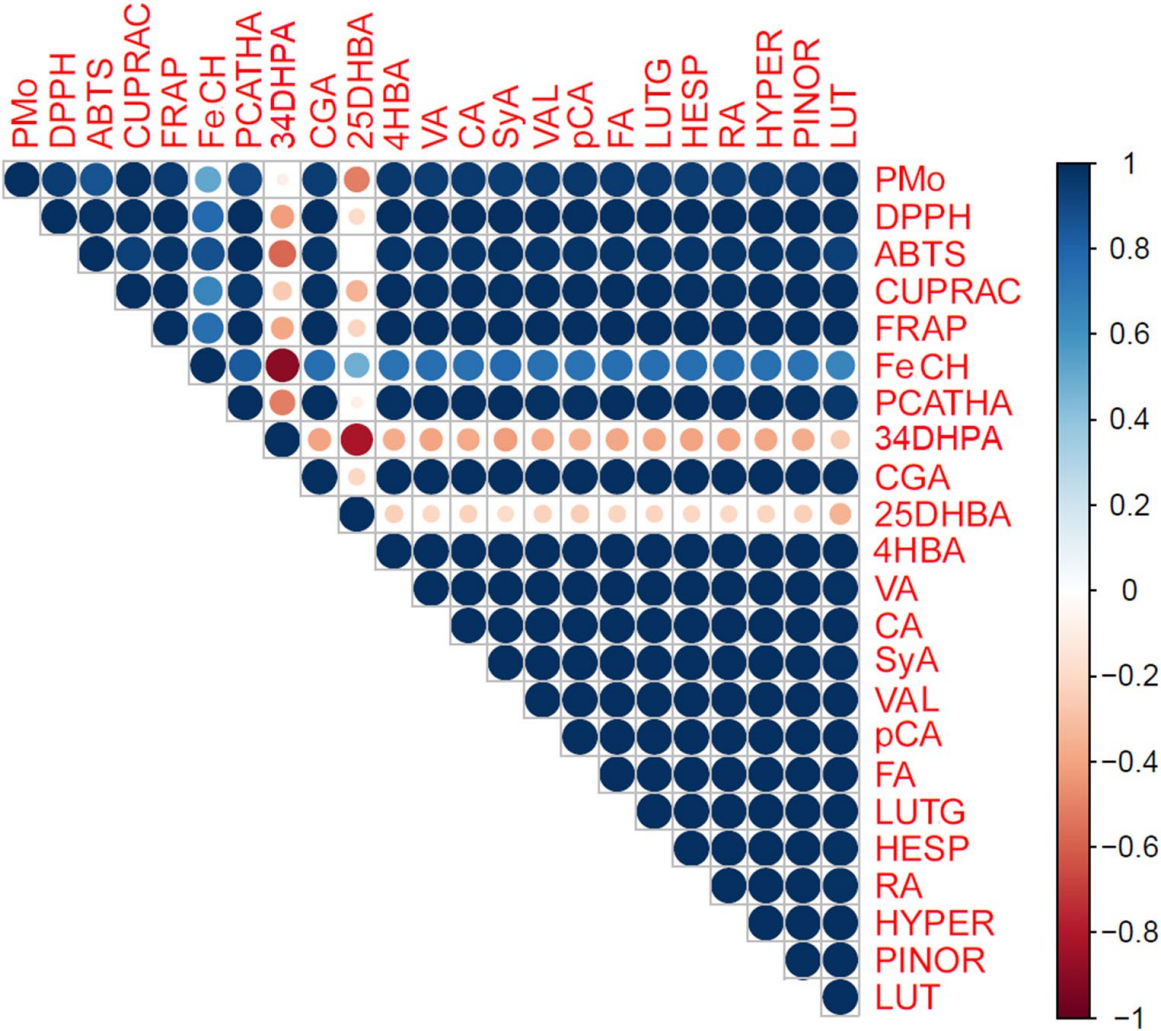
Correlation matrix between antioxidant activity and the most abundant phenolic compounds in the extracts of *Onosma riedliana* P-Mo, phosphomolybdenum assay; FeCH, ferrous ion chelating assay; PCATHA, protocatechuic acid; 34DHPA, 3,4-dihydroxyphenylacetic acid; CGA, chlorogenic acid; 25DHBA, 2,5-dihydroxybenzoic acid; 4HBA, 4-hydroxybenzoic acid; VA, vanillic acid; CA, caffeic acid; SyA, syringic acid; VAL, vanillin; pCA, *p*-coumaric acid; FA, ferulic acid; LUTG, luteolin-7-glucoside; HESP, hesperidin; RA, rosmarinic acid; HYPER, hyperoside; PINOR, pinoresinol; LUT, luteolin

The correlation matrix, presented in Fig. 2, illustrates the relationships between the antioxidant activity of three *O. riedliana* extracts, and the levels of the most abundant phenolic compounds (> 100.00 μg/g) present in these extracts. Remarkably, all the phenolic compounds, with the exception of 2,5-dihydroxybenzoic and 3,4-dihydroxyphenylacetic acids, exhibited notably high correlations with both the radical scavenging assays and the assays related to reducing stable cations. When considering correlation data from other *Onosma* species, it becomes evident that flavonoids tend to yield higher Pearson correlation coefficients compared to phenolic acids. This suggests that flavonoids are primarily responsible for the extracts’ ability to scavenge free radicals and reduce stable cations. Among these compounds, luteolin and its glycoside, hesperidin, as well as quercetin, kaempferol, and apigenin, play pivotal roles in conferring antioxidant properties to the extracts [6, 8, 11–13, 15]. Therefore, flavonoid-rich extracts of *O. riedliana* could be used in the treatment of different degenerative diseases, such as cardiovascular and inflammatory disease, cataracts, and cancer [9, 11, 15].

### Enzyme Inhibitory Activity

**Fig. 3:**
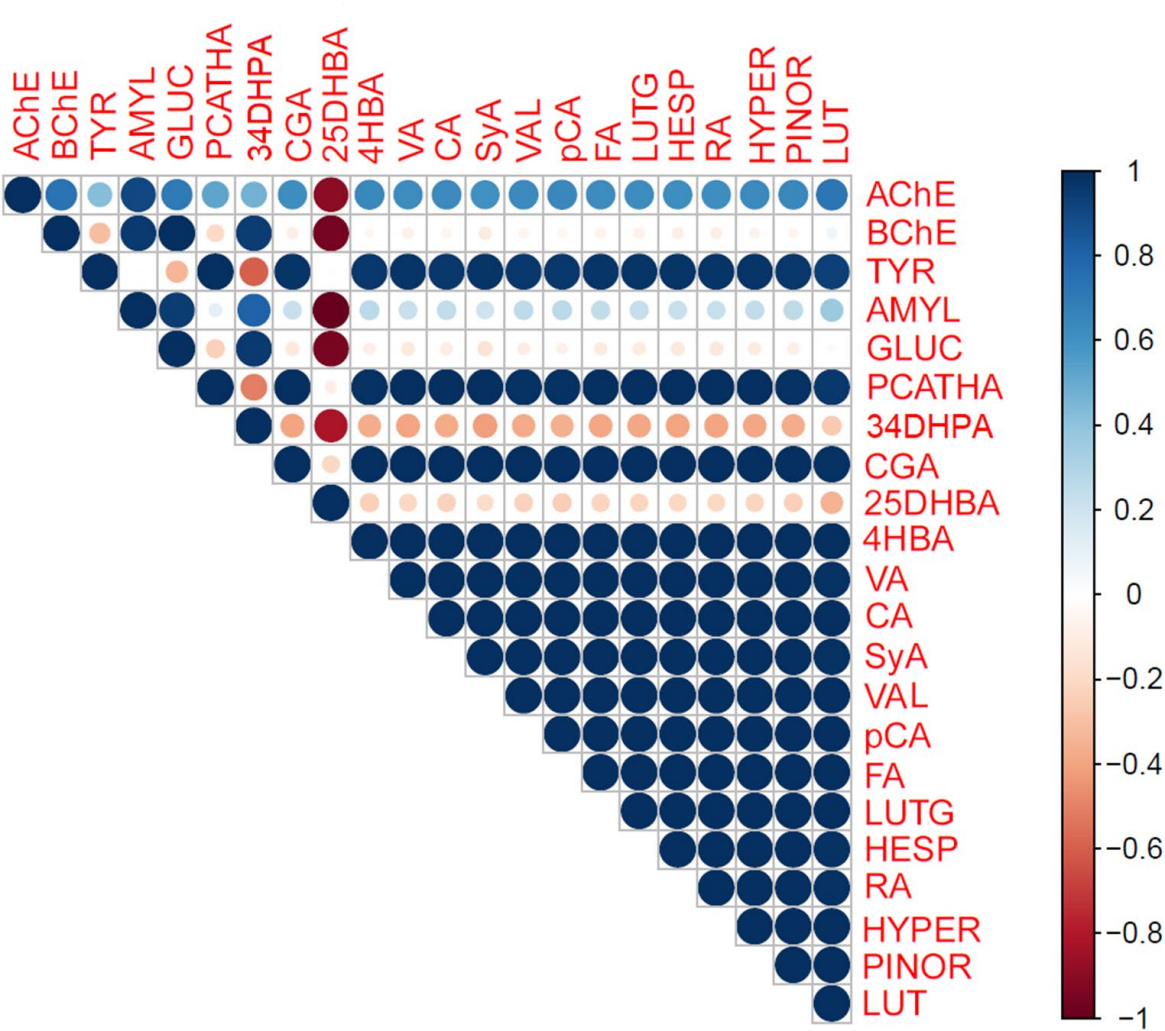
Correlation matrix between enzyme inhibitory activity and the most abundant phenolic compounds in the extracts of *Onosma riedliana* AChE, acetylcholinesterase inhibitory assay; BChE, butyrylcholinesterase inhibitory assay; TYR, tyrosinase inhibitory assay; AMYL, α-amylase inhibitory assay; GLUC, α‐glucosidase inhibitory assay; PCATHA, protocatechuic acid; 34DHPA, 3,4-dihydrohxyphenylacetic acid; CGA, chlorogenic acid; 25DHBA, 2,5-dihydroxybenzoic acid; 4HBA, 4-hydroxybenzoic acid; VA, vanillic acid; CA, caffeic acid; SyA, syringic acid; VAL, vanillin; pCA, *p*-coumaric acid; FA, ferulic acid; LUTG, luteolin-7-glucoside; HESP, hesperidin; RA, rosmarinic acid; HYPER, hyperoside; PINOR, pinoresinol; LUT, luteolin

The exploration of *O. riedliana*’s potential as a source of enzymatic inhibitors aligns with the growing interest in natural remedies for different disorders and the development of safer and more sustainable treatment options. The results presented in Table 3 highlight the differential inhibitory activities of the three *O. riedliana* extracts against various enzymes. The ethyl acetate extract exhibited the lowest IC_50_ values for the inhibition of cholinesterases, α-amylase, and α-glucosidase, while the methanolic extract showed the most favorable outcomes in inhibiting tyrosinase. Additionally, the results are expressed as equivalents of positive probes used in these assays, such as galanthamine (mg GAL/g), kojic acid (mg KA/g), and acarbose (mg AC/g). In this context, the ethyl acetate extract displayed the highest values, except for the inhibition of tyrosinase. Similar findings were reported for *O. tauricum* var. *tauricum* [8], *O. gigantea* [11], and *O. pulchra* [11], where ethyl acetate extracts demonstrated superior activities against these enzymes. The Pearson correlation matrix between enzyme inhibitory activities and the most abundant phenolic compounds in *O. riedliana* extracts (Fig. 3) suggests that all flavonoids, including luteolin, luteolin-7-glucoside, hesperidin, and hyperoside, as well as phenolic acids (excluding 3,4-dihydroxyphenylacetic and 2,5-dihydroxybenzoic acids), exhibit a highly significant correlation with the inhibitory activity of the extracts against the enzyme tyrosinase. Furthermore, the results indicate that phenolic compounds are primarily responsible for the inhibition of the enzyme acetylcholinesterase but not for butyrylcholinesterase or the two carbohydrate hydrolytic enzymes, α-amylase and α-glucosidase. These findings align with previous studies by Baltaci et al. [5] and Sarikukcu et al. [14], which conducted docking analyses and found that flavonoids like hesperidin and hyperoside had strong binding activity for both cholinesterases and tyrosinase, while their activities were comparatively lower for other enzymes. To summarize, phenolic-rich plant extracts of *O. riedliana* showed pharmacological potential as inhibitors of enzyme cholinesterase, which activity is related with neurodegenerative diseases. Also, these extract could be used as natural plant-based tyrosinase inhibitors, that normally are used for the prevention of severe skin diseases [9, 11, 15].

**Table 3:** Enzyme inhibitory activity (IC_50_: mg/mL) of *Onosma riedliana* extracts

Assay	Unit		Extract		Galanthamine	Kojic acid	Acarbose
		Ethyl acetate	Methanol	Water			
AChE	IC_50_ (mg/mL)	1.43 ± 0.02^b^	1.65 ± 0.04^b^	15.93 ± 0.96^c^	0.004 ± 0.000^a^		
	mg GAL/g	2.24 ± 0.05^a^	2.59 ± 0.03^a^	0.23 ± 0.01^b^			
BChE	IC_50_ (mg/mL)	5.39 ± 0.30^c^	2.29 ± 0.01^b^	na	0.004 ± 0.000^a^		
	mg GAL/g	1.53 ± 0.00^b^	0.65 ± 0.04^a^	na			
Tyrosinase	IC_50_ (mg/mL)	1.34 ± 0.01^b^	1.84 ± 0.03^d^	1.70 ± 0.02^c^		0.08 ± 0.00^a^	
	mg KA/g	41.53 ± 0.70^b^	57.16 ± 0.15^a^	45.10 ± 0.61^b^			
α-Amylase	IC_50_ (mg/mL)	1.75 ± 0.01^c^	1.26 ± 0.02^b^	17.97 ± 0.09^d^			0.96 ± 0.03^a^
	mg AC/g	754.11 ± 9.02^a^	542.07 ± 2.17^b^	52.86 ± 0.26^c^			
Glucosidase	IC_50_ (mg/mL)	2.38 ± 0.03^b^	1.09 ± 0.01^a^	10.40 ± 0.62^c^			1.65 ± 0.04^ab^
	mg AC/g	1538.87 ± 1.50^a^	702.75 ± 8.32^b^	160.85 ± 9.61^c^			

The values indicated by the same superscripts within the same row do not differ significantly after Tukey’s post hoc test at a 5% significance level; na, not active

## Conclusions

This paper presents the first-ever exploration of the phenolic profiles and biological activities of three extracts derived from *Onosma riedliana*, a plant belonging to a genus with a long history of therapeutic use for diverse disorders. The primary phenolic compounds identified in these extracts include lignan pinoresinol, flavonoid hesperidin, and phenolic acids, specifically 4-hydroxybenzoic and *p*-coumaric acids. The antioxidant potential of *Onosma riedliana* was assessed using six distinct assays. The methanolic extract displayed the highest antioxidant potency against oxidative agents, primarily attributed to the presence of flavonoids such as hesperidin and luteolin. Additionally, the ethyl acetate extract demonstrated notable inhibitory activity against two enzymes, cholinesterase and tyrosinase, while the methanolic extract exhibited remarkable efficacy against two carbohydrate hydrolytic enzymes, α-amylase and α-glucosidase. Statistical analysis of the data suggests that phenolic compounds, particularly flavonoids, are responsible for the inhibition of cholinesterases and tyrosinase, though they do not influence α-amylase and α-glucosidase. In conclusion, the findings presented herein underscore the potential of *Onosma riedliana* as a valuable source of bioactive natural products. Its diverse array of compounds highlights its suitability for various pharmaceutical formulations, indicating its significance in the realm of herbal medicine and natural product-based therapeutics, mainly in the treatment of different neurodegenerative diseases.

### Electronic Supplementary Material

Below is the link to the electronic supplementary material.

Supplementary Material 1

Supplementary Material 2

Supplementary Material 3

## Data Availability

The authors confirm that the data supporting the findings of this study are available within the article and its supplementary materials. Raw data are on request from the corresponding author.
