# Supplementary material for: Exploring the Pharmacological Potential of Onosma riedliana: Phenolic Compounds and Their Biological Activities
Source: Plant Foods Hum Nutr. 2023 Dec 16;79(1):106–12. doi: 10.1007/s11130-023-01131-0 (PMC10891197; doi:10.1007/s11130-023-01131-0)

**Exploring the Pharmacological Potential of *Onosma riedliana*: Phenolic Compounds and Their Biological Activities**

Sanja Ćavar Zeljkovıć^1,2^, Saliha Seyma Sahinler^3^, Cengiz Sarikurkcu^3^, Bulent Kirkan^3^, Riza Binzet^4^, Petr Tarkowski^1,2,*^

^1^ Czech Advanced Technology and Research Institute, Palacky University, Šlechtitelů 27, 78371 Olomouc, Czech Republic.

^2^ Centre of the Region Haná for Biotechnological and Agricultural Research, Department of Genetic Re-sources for Vegetables, Medicinal and Special Plants, Crop Research Institute, Šlechtitelů 29, 78371 Olomouc, Czech Republic.

^3^ Afyonkarahisar Health Sciences University, Faculty of Pharmacy, TR-03100, Afyonkarahisar-Turkey.

^4^ Mersin University, Faculty of Arts and Science, Department of Biology, TR-33343, Mersin-Turkey.

*Correspondence: petr.tarkowski@upol.cz

**Material and Methods**

***Chemical and reagents***

Gallic acid, (+)-catechin, pyrocatechol, chlorogenic acid, 2,5-dihydroxybenzoic acid, 4-hydroxybenzoic acid, (−)-epicatechin, caffeic acid, syringic acid, vanillin, taxifolin, sinapic acid, *p*-coumaric acid, ferulic acid, rosmarinic acid, 2-hydroxycinnamic acid, pinoresinol, quercetin, luteolin, and apigenin were purchased from Sigma-Aldrich (St. Louis, MO, USA). Vanillic acid, 3-hydroxybenzoic acid, 3,4-dihydroxyphenylacetic acid, apigenin 7-glucoside, luteolin 7-glucoside, hesperidin, eriodictyol, and kaempferol were obtained from Fluka (St. Louis, MO, USA). Finally, verbascoside, protocatechuic acid, and hyperoside were purchased from HWI Analytik (Ruelzheim, Germany). Methanol and formic acid of HPLC grade were purchased from Sigma-Aldrich (St. Louis, MO, USA) and Merck (Darmstadt, Germany), respectively. Ultra-pure water (18 mΩ) was obtained from a Milli-Q water purification system (Millipore Co., Ltd.). Ethyl acetate and methanol were obtained from Carlo Erba Reagents (Milan, Italy). Ultra-pure water was obtained using a Millipore Milli-Q Plus water treatment system (Millipore Bedford Corp., Bedford, MA).

*Plant material and extract preparation*

*Onosma riedliana* Binzet & Orcan was collected from the Gülnar to Ermenek 42 km, roadside, and open field, Mersin-Turkey (1300 m, 36° 26’ N 033° 06’ E) in August 2020 (Figure S1). The plants were identified and deposited by Dr. Riza Binzet from the Department of Biology, Mersin University, Mersin-Turkey. (Herbarium no: Binzet 202027).

The aerial parts of the plant were dried for five weeks in a dark and well-ventilated room. The dried material was then ground in a laboratory blender and then subjected to extraction. A 5 g of plant material was macerated with 100 ml of solvent for 24 hours to prepare ethyl acetate (EtOAc) and methanol (MeOH) extracts. Solvents were then removed under a vacuum. To obtain the water extract, the ground aerial parts were infused in boiling water for 15 min. After the extraction was complete, the mixture was lyophilized. The extracts were maintained at +4 °C until use, and they were prepared in the same concentration of 2 mg/mL.

***Determination of the phenolic and flavonoid content***

The total phenolic content in the plant extracts was determined by the method described before [1] with slight modification. Briefly, 0.25 mL of extract solution was mixed with 1 mL of Folin-Ciocalteu reagent (1:9) and shaken vigorously. After 3 min, 0.75 mL of 1 % Na_2_CO_3_ solution was added and the sample absorbance was read at 760 nm after 2 h incubation at room temperature. The total phenolic content was expressed as gallic acid equivalents (GAE) per gram of extract.

The total flavonoid content was determined according to the method described by Berk et al. [2]. Briefly, 1 mL of sample solution was mixed with the same volume of 2 % AlCl_3_ in methanol. Absorbances were read at 415 nm after 10 min incubation at room temperature. The total flavonoid content was expressed as rutin equivalents (RE) per gram of extract.

***UHPLC-MS/MS analysis of the extracts***

Chromatographic analysis of plant extracts was performed with an Agilent Technologies 1260 Infinity liquid chromatography system hyphenated with 6420 Triple Quad mass spectrometer. Separation of phenolic analytes was carried out on a Poroshell 120 EC-C18 (100 mm × 4.6 mm I.D., 2.7 μm) column, using 0.1% formic acid (component A) and methanol (component B) as the mobile phase. The gradient profile was set as follows: 0.00 to 3.00 min 2 % B, 6.00 min 25 % B, 10.00 min 50 % B, 14.00 min 95 % B, 17.00 min 95 % B, and 17.50 min 2 % B. The column temperature was maintained at 25°C. The flow rate was 0.4 mL/min and the injection volume was 2.0 μL. The mass spectrometer was inter-faced with the LC system via an ESI source. The electrospray source of the MS was operated in negative and positive multiple reaction monitoring (MRM) mode and the interface conditions were as follows: capillary voltage of −3.5 kV, gas temperature of 300°C, and gas flow of 11 L/min. The nebulizer pressure was 40 psi. More details about MRM conditions are listed in Supplementary table S1, while linearity and limits of detection and quantification are summarized in Supplementary table S2. Phenolic profiles of *O. riedliana* extracts are presented as LCMS chromatograms in Figure S2.

***Evaluation of the biological activity***

The antioxidant activity of the extracts was evaluated by phosphomolybdenum, 1,1-diphenyl-2-picrylhydrazyl (DPPH) radical scavenging activity, 2,2'-azino-bis(3-ethylbenzothiazoline-6-sulfonic acid) (ABTS) radical scavenging activity, ferrous chelating activity, ferric reducing antioxidant power (FRAP), and cupric ion reducing activity (CUPRAC) methods. All protocols are already described in the literature [3-5].

Furthermore, plant extracts were assayed for the inhibitory activities of the following enzymes: α-amylase, α-glucosidase, tyrosinase, acetylcholinesterase (AChE), and butyrylcholinesterase (BChE); according to the protocols already described in the literature [6].

***Statistical analysis***

All tests were carried out in triplicate. Tukey's test was used for the determination the degree of statistical difference. Because each of the antioxidant tests is based on a different mechanism of action results (e.g. radical scavenging, reducing power, chelating activity, etc.), RACI (relative antioxidant capacity index) values were determined to compare the superiority of the results obtained [25]. The correlation between RACI values and antioxidant activities of each sample was also determined. In addition, Pearson correlation analysis (by using SPSS v. 22.0) was performed to reveal the relationship of main phytochemical groups, phenolics, and flavonoids, with activity. Correlation matrices were constructed using RStudio (Version 2022.07.1.1. © 2009-2022 RStudio, Inc., Boston, MA, USA) using the corrplot package.

**References**

1. Slinkard K, Singleton VL (1977) Total phenol analyses: automation and comparison with manual methods. Am J Enol Vitic 28:49-55. https://www.ajevonline.org/content/28/1/49
2. Berk S, Tepe B, Arslan S, Sarikurkcu C (2011) Screening of the antioxidant, antimicrobial and DNA damage protection potentials of the aqueous extract of *Asplenium ceterach* DC. Af J Biotechnol 10:8902-8908. https://www.ajol.info/index.php/ajb/article/view/95603
3. Apak R, Güçlü K, Özyürek M, Esin Karademir S, Erçaǧ E (2006) The cupric ion reducing antioxidant capacity and polyphenolic content of some herbal teas. Int J Food Sci Nutr 57:92-304. https://doi.org/10.1080/09637480600798132
4. Kocak MS, Sarikurkcu C, Cengiz M, Kocak S, Uren MC, Tepe B (2016) *Salvia cadmica*: Phenolic composition and biological activity. Ind Crop Prod 85:204-212. https://doi.org/10.1016/j.indcrop.2016.03.015
5. Tepe B, Sarikurkcu C, Berk S, Alim A, Akpulat HA (2011) Chemical composition, radical scavenging and antimicrobial activity of the essential oils of *Thymus boveii* and *Thymus hyemalis*. Rec Nat Prod 5:208-220. https://acgpubs.org/article/records-of-natural-products/2011/3-july-september/chemical-composition-radical-scavenging-and-antimicrobial-activity-of-the-essential-oils-of-thymus-boveii-and-thymus-hyemalis
6. Zengin G, Sarikurkcu C, Gunes E, Uysal A, Ceylan R, Uysal S, Gungor H, Aktumsek A (2015) Two *Ganoderma* species: Profiling of phenolic compounds by HPLC-DAD, antioxidant, antimicrobial and inhibitory activities on key enzymes linked to diabetes mellitus, Alzheimer's disease and skin disorders. Food Funct 6:2794-2802. <https://doi.org/10.1039/c5fo00665a>

**Table S1.** MRM conditions for the analysis of phenolic compounds in plant extracts.

| Target compounds | Rt (min) | Precursor ion | MRM1 (CE, V) | MRM2 (CE, V) |
| --- | --- | --- | --- | --- |
| *Compounds analyzed by negative ionization mode [M-H]-* | | | | |
| Gallic acid | 8.891 | 168.9 | 125.0 (10) | – |
| Protocatechuic acid | 10.818 | 152.9 | 108.9 (12) | – |
| 3,4-Dihydroxyphenylacetic acid | 11.224 | 167.0 | 123.0 (2) | – |
| (+)-Catechin | 11.369 | 289.0 | 245.0 (6) | 202.9 (12) |
| Pyrocatechol | 11.506 | 109.0 | 90.6 (18) | 52.9 (16) |
| 2,5-Dihydroxybenzoic acid | 12.412 | 152.9 | 109.0 (10) | – |
| 4-Hydroxybenzoic acid | 12.439 | 136.9 | 93.1 (14) | – |
| Caffeic acid | 12.841 | 179.0 [ | 135.0 (12) | – |
| Vanillic acid | 12.843 | 166.9 | 151.8 (10) | 122.6 (6) |
| Syringic acid | 12.963 | 196.9 | 181.9 (8) | 152.8 (6) |
| 3-Hydroxybenzoic acid | 13.259 | 137.0 | 93.0 (6) | – |
| Vanillin | 13.397 | 151.0 | 136.0 (10) | – |
| Verbascoside | 13.589 | 623.0 | 461.0 (26) | 160.8 (36) |
| Taxifolin | 13.909 | 303.0 | 285.1 (2) | 125.0 (14) |
| Sinapic acid | 13.992 | 222.9 | 207.9 (6) | 163.8 (6) |
| p-Coumaric acid | 14.022 | 162.9 | 119.0 (12) | – |
| Ferulic acid | 14.120 | 193.0 | 177.8 (8) | 134.0 (12) |
| Luteolin 7-glucoside | 14.266 | 447.1 | 285.0 (24) | – |
| Rosmarinic acid | 14.600 | 359.0 | 196.9 (10) | 160.9 (10) |
| 2-Hydroxycinnamic acid | 15.031 | 162.9 | 119.1 (10) | – |
| Pinoresinol | 15.118 | 357.0 | 151.0 (12) | 135.7 (34) |
| Eriodictyol | 15.247 | 287.0 | 151.0 (4) | 134.9 (22) |
| Quercetin | 15.668 | 301.0 [ | 178.6 (10) | 151.0 (16) |
| Kaempferol | 16.236 | 285.0 | 242.8 (16) | 229.1 (18) |
| *Compounds analyzed by positive ionization mode [M+H]+* | | | | |
| Chlorogenic acid | 11.802 | 355.0 | 163.0 (10) | – |
| (−)-Epicatechin | 12.458 | 291.0 | 139.1 (12) | 122.9 (36) |
| Hesperidin | 14.412 | 611.1 | 449.2 (4) | 303.0 (20) |
| Hyperoside | 14.506 | 465.1 | 303.1 (8) | – |
| Apigenin 7-glucoside | 14.781 | 433.1 | 271.0 (18) | – |
| Luteolin | 15.923 | 287.0 | 153.1 (34) | 135.1 (36) |
| Apigenin | 16.382 | 271.0 | 153.0 (34) | 119.1 (36) |

Rt, retention time; CE, collision energy.

**Table S2.** Linear ranges and limits of detection and quantification for phenolic compounds analyzed in plant extracts.

|  | Linearity and sensitivity characteristics | | | |  |
| --- | --- | --- | --- | --- | --- |
| Compounds | Range  (μg/L) | Linear equation | R^2^ | LOD  (μg/L) | LOQ  (μg/L) |
| Gallic acid | 5–500 | y = 4.82x − 26.48 | 0.9988 | 1.46 | 4.88 |
| Protocatechuic acid | 2.5–500 | y = 5.65x − 9.99 | 0.9990 | 1.17 | 3.88 |
| 3,4-Dihydroxyphenylacetic acid | 5–500 | y = 5.13x − 12.39 | 0.9990 | 1.35 | 4.51 |
| (+)-Catechin | 10–500 | y = 1.45x + 1.95 | 0.9974 | 3.96 | 13.20 |
| Pyrocatechol | 25–400 | y = 0.11x − 0.52 | 0.9916 | 9.62 | 32.08 |
| Chlorogenic acid | 1–500 | y = 12.14x + 32.34 | 0.9995 | 0.55 | 1.82 |
| 2,5-Dihydroxybenzoic acid | 5–500 | y = 3.79x − 14.12 | 0.9980 | 2.12 | 7.08 |
| 4-Hydroxybenzoic acid | 5–500 | y = 7.62x + 22.79 | 0.9996 | 1.72 | 5.72 |
| (−)-Epicatechin | 5–500 | y = 9.11x − 9.99 | 0.9971 | 1.85 | 6.18 |
| Caffeic acid | 5–500 | y = 11.09x + 16.73 | 0.9997 | 3.15 | 10.50 |
| Vanillic acid | 10–500 | y = 0.49x − 1.61 | 0.9968 | 2.56 | 8.54 |
| Syringic acid | 10–500 | y = 0.74x − 1.54 | 0.9975 | 3.75 | 12.50 |
| 3-Hydroxybenzoic acid | 5–500 | y = 3.69x − 12.29 | 0.9991 | 1.86 | 6.20 |
| Vanillin | 50–500 | y = 2.02x + 135.49 | 0.9926 | 15.23 | 50.77 |
| Verbascoside | 2.5–500 | y = 8.59x − 28.05 | 0.9988 | 0.82 | 2.75 |
| Taxifolin | 5–500 | y = 12.32x + 9.98 | 0.9993 | 1.82 | 6.05 |
| Sinapic acid | 5–500 | y = 2.09x − 6.79 | 0.9974 | 2.64 | 8.78 |
| *p*-Coumaric acid | 5–500 | y = 17.51x + 53.73 | 0.9997 | 1.93 | 6.44 |
| Ferulic acid | 5–500 | y = 3.32x − 4.30 | 0.9992 | 1.43 | 4.76 |
| Luteolin 7-glucoside | 1–500 | y = 45.25x + 156.48 | 0.9996 | 0.45 | 1.51 |
| Hesperidin | 5–500 | y = 5.98x + 0.42 | 0.9993 | 1.73 | 5.77 |
| Hyperoside | 2.5–500 | y = 16.32x − 1.26 | 0.9998 | 0.99 | 3.31 |
| Rosmarinic acid | 1–500 | y = 9.82x − 17.98 | 0.9989 | 0.57 | 1.89 |
| Apigenin 7-glucoside | 1–500 | y = 21.33x − 31.69 | 0.9983 | 0.41 | 1.35 |
| 2-Hydroxycinnamic acid | 1–500 | y = 16.72x − 26.94 | 0.9996 | 0.61 | 2.03 |
| Pinoresinol | 10–500 | y = 0.80x − 2.69 | 0.9966 | 3.94 | 13.12 |
| Eriodictyol | 2.5–500 | y = 14.24x − 0.50 | 0.9998 | 0.80 | 2.68 |
| Quercetin | 5–500 | y = 14.68x − 18.25 | 0.9997 | 1.23 | 4.10 |
| Luteolin | 5–500 | y = 8.96x + 26.80 | 0.9992 | 1.34 | 4.46 |
| Kaempferol | 10–500 | y = 0.82x − 3.06 | 0.9959 | 3.30 | 10.99 |
| Apigenin | 2.5–500 | y = 11.29x + 38.05 | 0.9987 | 0.96 | 3.20 |

LOD, limit of detection; LOQ, limit of quantification.

Figure S1. *Onosma riedliana* Binzet & Orcan.


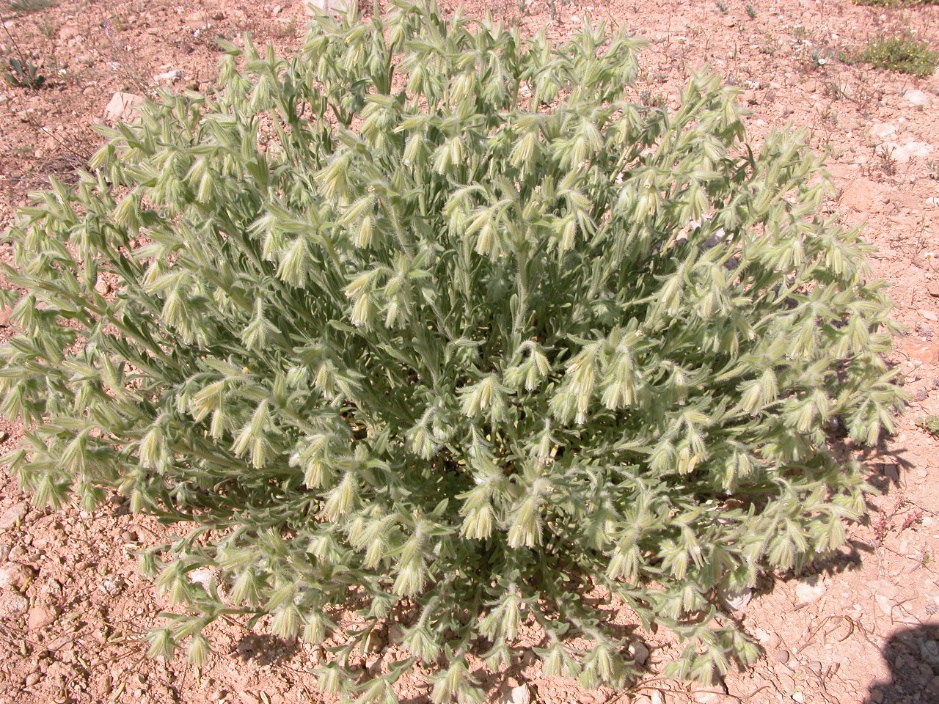


Figure S2. LCMS chromatograms of *O. riedliana* extracts.


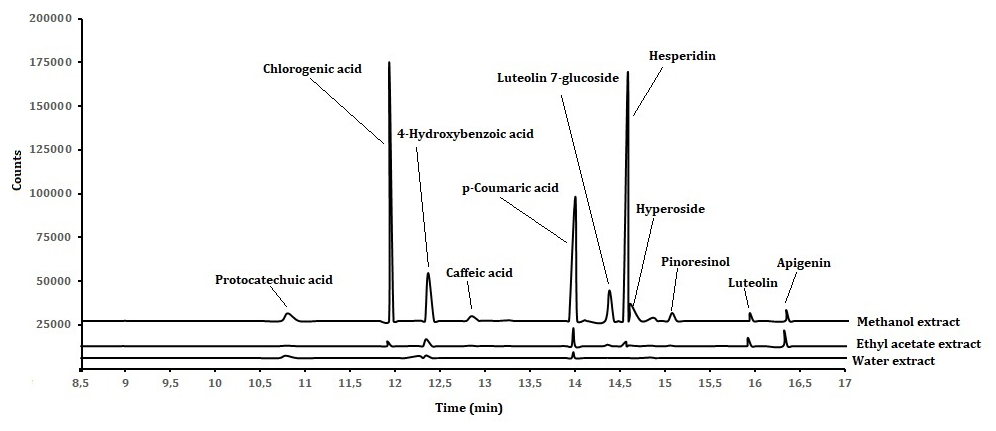

Supplement: Supplementary file 1 — Supplementary Material 1 [file 11130_2023_1131_MOESM1_ESM.docx]
