# Supplementary figures and images for: Exploring the Pharmacological Potential of Onosma riedliana: Phenolic Compounds and Their Biological Activities
Source: Plant Foods Hum Nutr. 2023 Dec 16;79(1):106–12. doi: 10.1007/s11130-023-01131-0 (PMC10891197; doi:10.1007/s11130-023-01131-0)

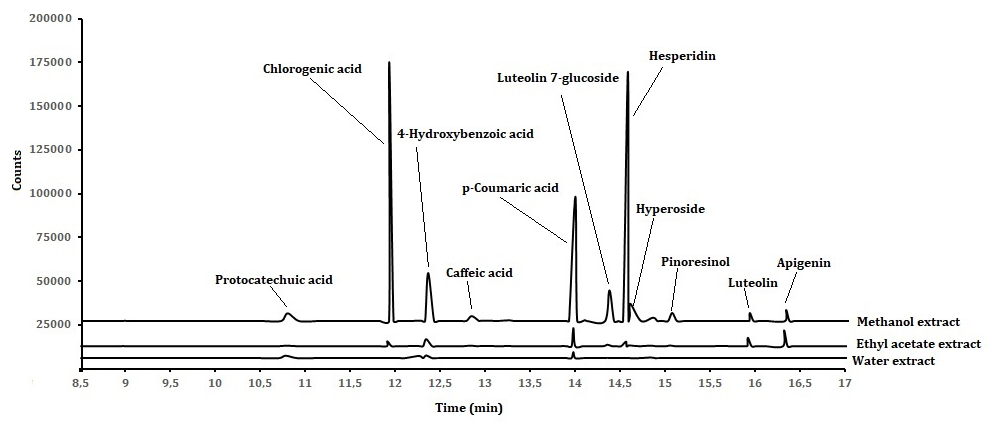

Supplement: Supplementary file 2 — Supplementary Material 2 [file 11130_2023_1131_MOESM2_ESM.png]

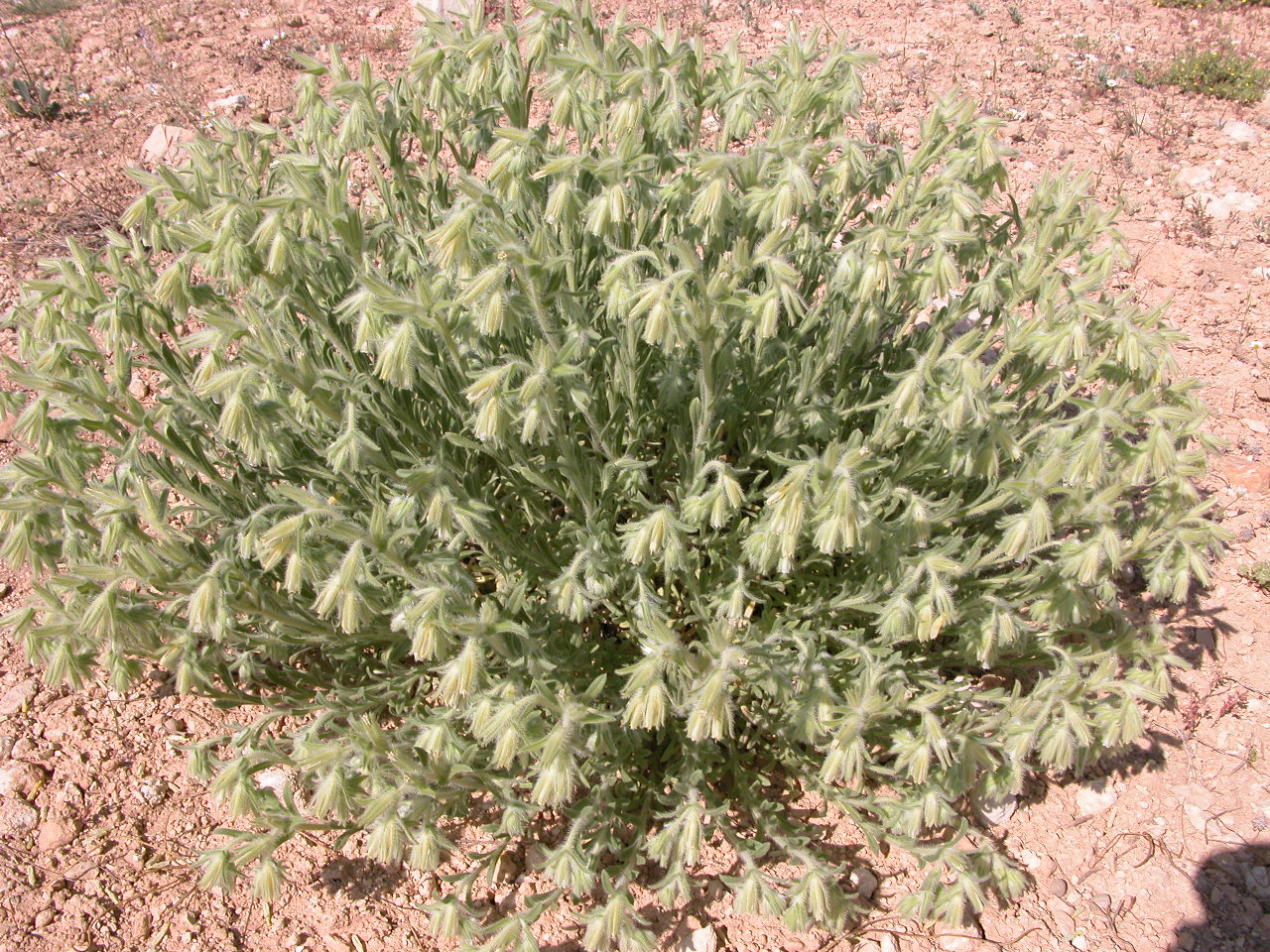

Supplement: Supplementary file 3 — Supplementary Material 3 [file 11130_2023_1131_MOESM3_ESM.png]
